# Performance evaluation of Automated Fluorescent Immunoassay System ROTA and NORO for detection of rotavirus and norovirus: A comparative study of assay performance with RIDASCREEN^®^ Rotavirus and Norovirus

**DOI:** 10.1002/jcla.23585

**Published:** 2020-09-23

**Authors:** Changhee Ha, In Young Yoo, Sun Ae Yun, Yoo Na Chung, Hee Jae Huh, Nam Yong Lee

**Affiliations:** ^1^ Department of Laboratory Medicine and Genetics Samsung Medical Center Sungkyunkwan University School of Medicine Seoul Korea; ^2^ Samsung Biomedical Research Institute Samsung Medical Center Sungkyunkwan University School of Medicine Seoul Korea

**Keywords:** AFIAS‐Noro, AFIAS‐Rota, immunoassay, norovirus, performance, rotavirus

## Abstract

**Background:**

The Automated Fluorescent Immunoassay System ROTA (AFIAS‐Rota) and NORO (AFIAS‐Noro) assays (Boditech Med Inc.) are newly developed diagnostic tests for rotavirus and norovirus infections.

**Methods:**

Performance of AFIAS‐Rota/Noro assays was evaluated in comparison with RIDASCREEN^®^ Rotavirus and Norovirus ELISA kits (R‐Biopharm) using clinical stool samples submitted from November 2018 to January 2019. Multiplex real‐time reverse transcription‐polymerase chain reaction was used as reference method.

**Results:**

A total of 256 clinical specimens were analyzed. AFIAS‐Rota and RIDASCREEN Rotavirus had almost perfect agreement (Kappa value = 0.95), and substantial agreement was observed between AFIAS‐Noro and RIDASCREEN Norovirus (Kappa value = 0.80). For detection of rotavirus, AFIAS and RIDASCREEN assays showed satisfactory diagnostic sensitivity (100% and 97.8%, respectively) and specificity (99.5% and 99.1%). For detection of norovirus, the RIDASCREEN assay showed significantly higher sensitivity than the AFIAS‐Noro (86.0% and 66.0%, respectively; *P* = .002). Analytic specificity of AFIAS‐Rota/Noro assays showed no cross‐reactivity against any other bacteria (14 strains) or viruses (2 strains). Hands‐on time (6 minutes) and turnaround time (26 minutes) required to perform AFIAS assays were much shorter than those required for RIDASCREEN assays (20 and 150 minutes, respectively).

**Conclusion:**

The AFIAS‐Rota/Noro assays showed overall excellent agreement with the RIDASCREEN assays. Although the AFIAS‐Noro assay exhibited lower sensitivity than the RIDASCREEN Norovirus assay for detection of norovirus, the AFIAS‐Rota/Noro assays could be useful as a rapid initial screening test in clinical laboratories due to its convenience and rapid turnaround time.

## 1 INTRODUCTION

Acute gastroenteritis is one of the most impactful and common infectious diseases, accounting for millions of deaths annually in young children. Rotavirus and norovirus are leading causes of acute viral gastroenteritis spread through fecal to oral transmission.^1^ Rotavirus infections are the primary cause of severe dehydrating gastroenteritis, especially in children below the age of five years.^2^ Norovirus is the single most common cause of acute gastroenteritis in adults and the second major cause of severe diarrhea in infants and young children in the United States.^3^ Patients with gastroenteritis are treated mainly with oral or intravenous rehydration solutions, and antibiotics are not routinely indicated in viral gastroenteritis.^1^ Accurate test results are beneficial in managing patients, such as isolating patients to prevent transmission and prompting physicians to consider antibiotics therapy.

For appropriate treatment and infection control, accurate and timely identification of pathogens is necessary. Various diagnostic tools including electron microscopy, latex agglutination, immunochromatographic assay (ICA), enzyme immunoassays, and molecular assays have been developed.^4^ Molecular methods such as reverse transcription‐polymerase chain reaction (RT‐PCR) are highly sensitive and specific. However, they are expensive and require specialized techniques and equipment.^4^, ^5^ On the other hand, ICA can be run individually, and ELISA assays can be easily performed without sophisticated equipment.^4^, ^6^ Thus, due to simplicity and swiftness, immunoassays including the ICA and enzyme‐linked immunosorbent assay (ELISA) have been commercially used in routine clinical laboratories.^5^

Here, we aimed to evaluate the performance of Automated Fluorescent Immunoassay System ROTA (AFIAS‐Rota) and NORO (AFIAS‐Noro) assays (Boditech Med Inc.), newly developed automated fluorescent lateral flow immunoassays, in comparison with RIDASCREEN^®^ Rotavirus (RIDASCREEN‐Rota) and Norovirus (RIDASCREEN‐Noro) ELISA kits (R‐Biopharm) for detection of rotavirus and norovirus.

## 2 MATERIALS AND METHODS

We used a total of 256 clinical stool samples submitted to the clinical microbiology laboratory at a tertiary referral hospital, from November 2018 to January 2019. After routine testing with multiplex real‐time RT‐PCR (rRT‐PCR), residual stool samples were stored at −70°C prior to analysis. This study was approved by the Institutional Review Board of Samsung Medical Center, Seoul, Korea (approval number 2018‐08‐110‐003).

AFIAS‐Rota and Noro assays were performed according to the manufacturer's instructions. Briefly, 50 microliters (μL) of diluted stool was added to the AFIAS cartridge sample well (Boditech Med Inc.), and results were read after 12 minutes (min) using an AFIAS‐6 scanner (Boditech Med Inc.). The scanner measured fluorescence intensity in the form of a relative cutoff index (COI) that was proportional to the concentration of the target antigens in the samples. The sample results were interpreted as “positive” when the COI of the AFIAS assays was ≥1.0, “negative” when COI was <0.9, or “indeterminate” when 0.9 ≤COI <1.0.^7^, ^8^

As a comparative method, rotavirus and norovirus antigen assays were performed using RIDASCREEN‐Rota and RIDASCREEN‐Noro assays according to the manufacturer's instructions. A total of 100 μL of stool with biotinylated anti‐rotavirus and norovirus antibodies were transferred to each sample well and incubated for 60 minutes at room temperature. After washing with washing buffer five times, streptavidin poly‐peroxidase conjugates were added and incubated for 30 minutes. After washing, the substrates were added, followed by a 15‐minute incubation period and addition of a stop reagent. The fluorescence was analyzed using a GEMINI spectrofluorometer (STRATEC Biomedical AG).

Multiplex rRT‐PCR assays were performed as a reference method using the PowerChek™ Adeno/Astro/Rotavirus and PowerChek™ Norovirus GI/GII Real‐time PCR Kits (Kogene Biotech). Briefly, viral ribonucleic acid (RNA) was extracted from stool samples on a MagNA Pure 96 nucleic extraction system (Roche Diagnostics) according to the manufacturer's recommendations. rRT‐PCR was performed for a total volume of 20 μL (15 μL PCR mixture and 5 μL template RNA) using the ABI 7500 fast real‐time PCR system (Applied Biosystems). All procedures were performed according to the manufacturer's instructions.

Positive percent agreement (PPA), negative percent agreement (NPA), kappa coefficient, and their 95% confidence interval (CI) were calculated to compare agreement between AFIAS and RIDASCREEN for detection of rotavirus and norovirus. The diagnostic sensitivity and specificity of the AFIAS and RIDASCREEN assays were calculated against the results of rRT‐PCR. McNemar's test was used to compare sensitivity and specificity between AFIAS and RIDASCREEN for rotavirus and norovirus. Statistical analyses were performed using MedCalc Statistical Software version 19.0.5 (MedCalc Software, Ostend, Belgium) and the VassarStats website (http://vassarstats.net/).

Analytical sensitivity of the AFIAS assay was determined using rotavirus and norovirus reference materials obtained from Korean National Biological Reference Standard. Probit analysis was used to determine the 95% cutoff value, and serial dilutions were analyzed with eight replicates per dilution.^9^ Analytical specificity was evaluated using 16 reference or clinical bacterial and viral strains including commonly isolated bacteria/viruses from stool.

Hands‐on time (HOT) and turnaround time (TAT) of the AFIAS and RIDASCREEN assays were measured for workflow analysis. Since the AFIAS‐6 scanner has six channels, the HOT and TAT of the two assays were measured for six samples. HOT was defined as the time spent by a trained laboratory technician for preparing samples prior to equipment loading and detection. TAT was defined as the time interval between laboratory receipt of the sample and generation of the final result.

## 3 RESULT

Of 256 clinical stool samples, 46 were positive on both the AFIAS‐Rota and RIDASCREEN‐Rota assays (Table 1). The AFIAS‐Rota results were concordant with those of RIDASCREEN‐Rota for 252/256 (98.4%) samples, showing almost perfect agreement between the two rotavirus assays. Calculated PPA, NPA, and kappa value were 95.7% (95% CI, 84.0‐99.2), 99.0% (95% CI, 96.2‐99.8), and 0.95 (95% CI, 0.90‐1.00), respectively (Table 1). A total of 45 samples were rotavirus‐positive based on the reference method. As shown in Table 2, both assays exhibited excellent sensitivity and specificity. Diagnostic sensitivity of AFIAS‐Rota and RIDASCREEN‐Rota was 100% (95% CI, 90.2%‐100%) and 97.8% (95% CI, 86.8%‐99.9%), respectively. Diagnostic specificity of AFIAS‐Rota and RIDASCREEN‐Rota was 99.5% (95% CI, 97.0%‐100%) and 99.1% (95% CI, 96.3‐99.8), respectively.

**Table 1:** Comparison of the AFIAS and RIDASCREEN assays for detecting rotavirus and norovirus

Target	Method		RIDASCREEN			Agreement, % (95% CI)
			Positive	Negative	Total	
Rotavirus	AFIAS‐Rota	Positive	44	2	46	Positive percent agreement = 95.7% (95% CI, 84.0‐99.2)
		Negative	2	208	210	Negative percent agreement = 99.0% (95% CI, 96.2‐99.8)
		Total	46	210	256	Kappa value = 0.95 (95% CI, 0.90‐1.00)
Norovirus	AFIAS‐Noro	Positive	35	3	38	Positive percent agreement = 76.1% (95% CI, 60.9‐86.9)
		Negative	11	207	218	Negative percent agreement = 98.6% (95% CI, 95.5‐99.6)
		Total	46	210	256	Kappa value = 0.80 (95% CI, 0.70‐0.90)

Abbreviations: AFIAS, Automated Fluorescent Immunoassay System; CI, confidence interval.

**Table 2:** Performance of the AFIAS and RIDASCREEN assays for rotavirus and norovirus detection

Target	Method	Sensitivity	Specificity
		N/Total N	N/Total N
		% (95% CI)	% (95% CI)
Rotavirus	AFIAS‐Rota	45/45	210/211
		100% (90.2%‐100%)	99.5% (97.0%‐100%)
	RIDASCREEN‐Rota	44/45	209/211
		97.8% (86.8%‐99.9%)	99.1% (96.3%‐99.8%)
Norovirus	AFIAS‐Noro	33/50	201/206
		66.0% (51.1%‐78.4%)	97.6% (94.1%‐99.1%)
	RIDASCREEN‐Noro	43/50	203/206
		86.0% (72.6%‐93.7%)	98.5% (95.5%‐99.6%)

Abbreviations: AFIAS, Automated Fluorescent Immunoassay System; CI, confidence interval.

For norovirus detection, 38 and 46 samples were positive by the AFIAS‐Noro and RIDASCREEN‐Noro assays, respectively (Table 1). The AFIAS‐Noro results were concordant with those of RIDASCREEN‐Noro for 242/256 (94.5%) samples. PPA, NPA, and kappa value were 76.1% (95% CI, 60.9‐86.9), 98.6% (95% CI, 95.5‐99.6), and 0.80 (95% CI, 0.70‐0.90), respectively (Table 1). Diagnostic sensitivity of AFIAS‐Noro and RIDASCREEN‐Noro was 66.0 (95% CI, 51.1%‐78.4%) and 86.0% (95% CI, 72.6%‐93.7%), respectively. Diagnostic specificity of AFIAS‐Rota and RIDASCREEN‐Rota was 97.6% (95% CI, 94.1%‐99.1%) and 98.5% (95% CI, 95.5‐99.6), respectively (Table 2). Discordant results between the two assays were observed for 14 samples. A total of 11 results were positive by RIDASCREEN‐Noro but negative by the AFIAS‐Noro assay; 10 were confirmed to be positive by rRT‐PCR. Although the two assays showed comparable specificity, the sensitivity of the RIDASCREEN‐Noro assay was significantly higher than that of the AFIAS‐Noro assay (*P* = .002).

The 95% limit of detection was 2.3 × 10^6^ plaque‐forming units (PFU)/L (95% CI, 1.5 × 10^6^‐1.1 × 10^7^ PFU/L) and 0.7 mg/L (95% CI; 0.5‐8.0 mg/L) for rotavirus and norovirus, respectively. No cross‐reactivity was observed against any of the 14 bacteria or two viruses (Table 3).

**Table 3:** Analytic specificity of AFIAS assay

Microbial species	AFIAS‐Rota result (COI)		AFIAS‐Noro result (COI)	
	1st	2nd	1st	2nd
*Campylobacter jejuni*	Negative (0.21)	Negative (0.14)	Negative (0.35)	Negative (0.34)
*Enterobacter cloacae*	Negative (0.16)	Negative (0.16)	Negative (0.32)	Negative (0.29)
*Enterococcus faecalis*	Negative (0.13)	Negative (0.10)	Negative (0.34)	Negative (0.25)
*Escherichia coli*	Negative (0.16)	Negative (0.17)	Negative (0.33)	Negative (0.30)
*Proteus mirabilis*	Negative (0.17)	Negative (0.19)	Negative (0.39)	Negative (0.45)
*Pseudomonas aeruginosa*	Negative (0.21)	Negative (0.21)	Negative (0.35)	Negative (0.29)
*Salmonella enterica*	Negative (0.15)	Negative (0.13)	Negative (0.32)	Negative (0.37)
*Shigella sonnei*	Negative (0.19)	Negative (0.18)	Negative (0.32)	Negative (0.36)
*Yersinia enterocolitica*	Negative (0.25)	Negative (0.09)	Negative (0.36)	Negative (0.36)
*Streptococcus dysgalactiae*	Negative (0.01)	Negative (0.14)	Negative (0.38)	Negative (0.35)
*Clostridium difficile*	Negative (0.15)	Negative (0.19)	Negative (0.37)	Negative (0.39)
*Candida albicans*	Negative (0.17)	Negative (0.17)	Negative (0.36)	Negative (0.32)
*Citrobacter freundii*	Negative (0.11)	Negative (0.21)	Negative (0.34)	Negative (0.32)
*Vibrio parahaemolyticus*	Negative (0.21)	Negative (0.10)	Negative (0.31)	Negative (0.30)
Astrovirus	Negative (0.07)	Negative (0.19)	Negative (0.27)	Negative (0.30)
Adenovirus	Negative (0.12)	Negative (0.14)	Negative (0.60)	Negative (0.30)

Abbreviations: AFIAS, Automated Fluorescent Immunoassay System; COI, cutoff index.

**Figure 1:**
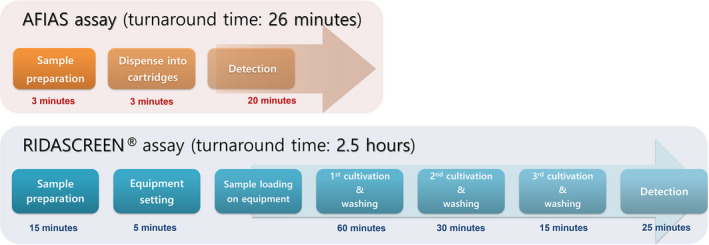
Comparison of workflow analysis between Automated Fluorescent Immunoassay System (AFIAS) and RIDASCREEN assays for performing six specimens

The HOT (6 minutes) and TAT (26 minutes) required to perform the AFIAS assay were much shorter than those required for the RIDASCREEN assay (HOT, 20 minutes; TAT, 150 minutes). The time difference between the two assays originated primarily from cultivation time in the RIDASCREEN assay. Workflow of the major steps in the two assays is illustrated in Figure 1.

## 4 DISCUSSION

In this study, we evaluated AFIAS‐Rota/Noro assays for detection of rotavirus and norovirus compared to the RIDASCREEN assays. For rotavirus, the AFIAS‐Rota assay and RIDASCREEN‐Rota assay were in good agreement and yielded satisfactory sensitivity (100% and 97.8%, respectively) and specificity (99.5% and 99.1%) compared to the rRT‐PCR assay result. These results were comparable to other published studies for detection of rotavirus.^6^, ^10^, ^11^ When using ICA, Kim et al^6^ showed sensitivity 100%, specificity 94.9%, and overall percent agreement (OPA) 95.6% for rotavirus, while Nguyen et al^10^ showed sensitivity 87.8%, specificity 93.3%, and OPA 89.4% for rotavirus.

However, the AFIAS‐Noro and RIDASCREEN‐Noro assays in our study showed relatively low sensitivity (66.0% and 86.0%, respectively) for norovirus detection. Likewise, several previously published reports with evaluation of ICA for detection of norovirus showed sensitivity values ranging from 57.1% to 76.5%, while specificity values varied from 92.5% to 100%.^12^, ^13^, ^14^, ^15^ Also, studies for detection of norovirus using ELISA tests showed sensitivity values widely varying from 31.6% to 83.8%, while specificity values varied from 92.5% to 98.7%.^16^, ^17^, ^18^ A total of 17 stool samples yielded negative results with the AFIAS‐Noro assay but tested positive using rRT‐PCR. Failure to detect norovirus by the AFIAS assay could be explained by the diversity of norovirus genotypes examined^16^, ^19^ or the difference in analytical sensitivity among the assays.^20^ Therefore, the AFIAS‐Noro is recommended as an initial screening test. Patients clinically suspicious for norovirus infection despite initial AFIAS‐negative results were indicated for reflex testing by molecular methods such as rRT‐PCR. Nevertheless, AFIAS‐Noro can be considered a useful initial screening test due to its simplicity and short TAT.

In conclusion, our data indicate that the AFIAS‐Rota/Noro assays show overall excellent agreement with the RIDASCREEN‐Rota/Noro assays. Although the AFIAS‐Noro assay exhibited lower sensitivity than the RIDASCREEN‐Noro assay for detection of norovirus, the AFIAS‐Rota/Noro assays could be useful as a rapid initial screening test in routine clinical laboratories due to their convenience and rapid TAT.

## AUTHOR CONTRIBUTIONS

Lee NY and Huh HJ initiated and designed the study and coordinated the drafting of the manuscript. Yun SA participated in specimen collection and experiments. Ha C and Yoo IY carried out data analysis and wrote the manuscript. Lee NY and Huh HJ supervised the study design and reviewed the manuscript. All authors read and approved the final manuscript.

## References

[1] Elliott EJ . Acute gastroenteritis in children. BMJ. 2007;334:35‐40. 17204802 10.1136/bmj.39036.406169.80 PMC1764079

[2] Crawford SE , Ramani S , Tate JE , et al. Rotavirus infection. Nat Rev Dis Primers. 2017;3:17083. 29119972 10.1038/nrdp.2017.83 PMC5858916

[3] Bok K , Green KY . Norovirus gastroenteritis in immunocompromised patients. N Engl J Med. 2012;367:2126‐2132. 23190223 10.1056/NEJMra1207742 PMC4944753

[4] de Bruin E , Duizer E , Vennema H , Koopmans MP . Diagnosis of Norovirus outbreaks by commercial ELISA or RT‐PCR. J Virol Methods. 2006;137:259‐264. 16901556 10.1016/j.jviromet.2006.06.024

[5] Kirby A , Gurgel RQ , Dove W , Vieira SC , Cunliffe NA , Cuevas LE . An evaluation of the RIDASCREEN and IDEIA enzyme immunoassays and the RIDAQUICK immunochromatographic test for the detection of norovirus in faecal specimens. J Clin Virol. 2010;49:254‐257. 20864394 10.1016/j.jcv.2010.08.004

[6] Kim J , Kim HS , Kim HS , et al. Evaluation of an immunochromatographic assay for the rapid and simultaneous detection of rotavirus and adenovirus in stool samples. Ann Lab Med. 2014;34:216‐222. 24790909 10.3343/alm.2014.34.3.216 PMC3999320

[7] Kim JS , Lee SK , Ko DH , Hyun J , Kim HS . Performance evaluation of the automated fluorescent immunoassay system rotavirus assay in clinical samples. Ann Lab Med. 2019;39:50‐57. 30215230 10.3343/alm.2019.39.1.50 PMC6143470

[8] Ryu JH , Kwon M , Moon JD , et al. Development of a rapid automated fluorescent lateral flow immunoassay to detect Hepatitis B Surface Antigen (HBsAg), Antibody to HBsAg, and antibody to Hepatitis C. Ann Lab Med. 2018;38:578‐584. 30027702 10.3343/alm.2018.38.6.578 PMC6056386

[9] Clinical Laboratory Standards Institute . Evaluation of Detection Capability for Clinical Laboratory Measurement Procedures, 2nd ed. CLSI document No. MM17. Wayne, PA: Clinical Laboratory Standards Institute; 2018.

[10] Nguyen TA , Khamrin P , Takanashi S , et al. Evaluation of immunochromatography tests for detection of rotavirus and norovirus among Vietnamese children with acute gastroenteritis and the emergence of a novel norovirus GII.4 variant. J Trop Pediatr. 2007;53:264‐269. 17496324 10.1093/tropej/fmm021

[11] De Grazia S , Bonura F , Pepe A , et al. Performance analysis of two immunochromatographic assays for the diagnosis of rotavirus infection. J Virol Methods. 2017;243:50‐54. 28159668 10.1016/j.jviromet.2017.01.025

[12] Takanashi S , Okame M , Shiota T , et al. Development of a rapid immunochromatographic test for noroviruses genogroups I and II. J Virol Methods. 2008;148:1‐8. 18054091 10.1016/j.jviromet.2007.10.010

[13] Park KS , Baek KA , Kim DU , et al. Evaluation of a new immunochromatographic assay kit for the rapid detection of norovirus in fecal specimens. Ann Lab Med. 2012;32:79‐81. 22259783 10.3343/alm.2012.32.1.79 PMC3255496

[14] Bruins MJ , Wolfhagen MJ , Schirm J , Ruijs GJ . Evaluation of a rapid immunochromatographic test for the detection of norovirus in stool samples. Eur J Clin Microbiol Infect Dis. 2010;29:741‐743. 20306322 10.1007/s10096-010-0911-5

[15] Battaglioli G , Nazarian EJ , Lamson D , Musser KA , St GK . Evaluation of the RIDAQuick norovirus immunochromatographic test kit. J Clin Virol. 2012;53:262‐264. 22226980 10.1016/j.jcv.2011.12.007

[16] Morillo SG , Luchs A , Cilli A , et al. Norovirus 3rd Generation kit: an improvement for rapid diagnosis of sporadic gastroenteritis cases and valuable for outbreak detection. J Virol Methods. 2011;173:13‐16. 21192981 10.1016/j.jviromet.2010.12.017

[17] Kim HS , Kim KH , Kwon HW , et al. Evaluation of Rapid Antigen Test for the Detection of Norovirus Infection: Comparison with ELISA and Real Time Quantitative Reverse Transcription PCR Assays. Lab Med Online. 2011;1:184‐189.

[18] Gray JJ , Kohli E , Ruggeri FM , et al. European multicenter evaluation of commercial enzyme immunoassays for detecting norovirus antigen in fecal samples. Clin Vaccine Immunol. 2007;14:1349‐1355. 17715333 10.1128/CVI.00214-07 PMC2168115

[19] Burton‐MacLeod JA , Kane EM , Beard RS , Hadley LA , Glass RI , Ando T . Evaluation and comparison of two commercial enzyme‐linked immunosorbent assay kits for detection of antigenically diverse human noroviruses in stool samples. J Clin Microbiol. 2004;42:2587‐2595. 15184438 10.1128/JCM.42.6.2587-2595.2004 PMC427881

[20] Kim HS , Kim JS . Discrepancies between antigen and polymerase chain reaction tests for the detection of rotavirus and norovirus. Ann Clin Lab Sci. 2016;46:282‐285. 27312553

